# Which is the best method of sterilization for recycled bone autograft in limb salvage surgery: a radiological, biomechanical and histopathological study in rabbit

**DOI:** 10.1186/s12885-015-1234-9

**Published:** 2015-04-15

**Authors:** Nor Faissal Yasin, Vivek Ajit Singh, Marniza Saad, Effat Omar

**Affiliations:** 1 Department of Orthopaedic Surgery, Faculty of Medicine, Universiti Teknologi MARA, Sungai Buloh Campus, Selangor, Malaysia; 2 Department of Orthopaedic Surgery, Faculty of Medicine, University of Malaya, Kuala Lumpur, Malaysia; 3 Department of Oncology, Faculty of Medicine, University of Malaya, Kuala Lumpur, Malaysia; 4 Department of Pathology, Faculty of Medicine, Universiti Teknologi MARA, Sungai Buloh Campus, Selangor, Malaysia

**Keywords:** Recycled bone autografts, Sterilization, Limb salvage surgery

## Abstract

**Background:**

Limb salvage surgery is a treatment of choice for sarcomas of the extremities. One of the options in skeletal reconstruction after tumour resection is by using a recycled bone autograft. The present accepted methods of recycling bone autografts include autoclaving, pasteurization and irradiation. At the moment there is lack of studies that compare the effectiveness of various sterilization methods used for recycling bone autografts and their effects in terms of bone incorporation. This study was performed to determine the effects of different methods of sterilization on bone autografts in rabbit by radiological, biomechanical and histopathological evaluations.

**Methods:**

Fresh rabbit cortical bone is harvested from the tibial diaphysis and sterilized extracorporeally by pasteurization (n = 6), autoclaving (n = 6), irradiation (n = 6) and normal saline as control group (n = 6). The cortical bones were immediately reimplanted after the sterilization process. The subsequent process of graft incorporation was examined over a period of 12 weeks by serial radiographs, biomechanical and histopathological evaluations. Statistical analysis (ANOVA) was performed on these results. Significance level (**α**) and power (**β**) were set to 0.05 and 0.90, respectively.

**Results:**

Radiographic analysis showed that irradiation group has higher score in bony union compared to other sterilization groups (p = 0.041). ANOVA analysis of ‘failure stress’, ‘modulus’ and ‘strain to failure’ demonstrated no significant differences (p = 0.389) between treated and untreated specimens under mechanical loading. In macroscopic histopathological analysis, the irradiated group has the highest percentage of bony union (91.7 percent). However in microscopic analysis of union, the pasteurization group has significantly higher score (p = 0.041) in callus formation, osteocytes percentage and bone marrow cellularity at the end of the study indicating good union potential.

**Conclusions:**

This experimental study shown that both irradiation and pasteurization techniques have more favourable outcome in terms of bony union based on radiographic and histopathological evaluations. Autoclaving has the worst outcome. These results indicate that extracorporeal irradiation or pasteurization of bone autografts, are viable option for recycling bone autografts. However, pasteurization has the best overall outcomes because of its osteocytes preservation and bone marrow cellularity.

## Background

Limb salvage surgery has all but replaced amputation as the treatment of choice for musculoskeletal sarcomas of the extremities. In the majority of cases, there is still an amputation rate of about 8%. This dramatic change came about as the result of better understanding of the tumour biology, better imaging modalities, more effective chemotherapy, improved radiotherapy techniques, a better characterization of the biomechanics of human skeleton, continuous refinement in surgical techniques, advances in material engineering and manufacturing techniques, and the development of a reliable, stable modular prosthesis for surgical reconstruction. Today, up to 95% of patients with osteosarcoma can be treated with limb-sparing surgery at major centres specializing in musculoskeletal oncology, and producing a dramatic improvement of long-term survival rate in 60-80% of patients with localized disease since the 1970s after the introduction of intensive multiagent adjuvant chemotherapy [1,2]. This is mainly due to a well—coordinated multidisciplinary approach involving different specialties. Neoadjuvant and adjuvant chemotherapy has been proven to improve the survival rate of patient with nonmetastatic and metastatic osteosarcoma.

The main aim of reconstruction surgery after oncologic resection include providing skeletal stability, adequate wound coverage to allow subsequent adjuvant therapy, restoration of acceptable functional capability and desirable aesthetic outcome when possible. Reconstruction of bone defect after completion of the tumour resection depends on the surgeon experience and available resources in the institution. Generally, small bone defect can be reconstructed with bone autograft from the patient’s own iliac crest or fibular grafts, but this is not possible in bigger defects as the amount of autograft available is limited. In these cases, the options of skeletal reconstruction include, either by a large endoprosthesis, bone allograft, a composite allograft and prosthesis, or recycled autograft.

The prospects of using patients own bone is appealing as it’s a form of biological reconstruction with identical anatomical match especially in large skeletal defect in pelvic sarcoma. The use of recycled bone autograft is controversial because of the concerns in the adequacy of tumour eradication prior to reimplantation. This has been studied by Singh VA et al [3], which showed satisfactory tumour cells eradication by using pasteurization and irradiation. The recycled bone graft has been used as an alternative to allograft mainly in Asian countries where there is limited bone banking facilities due to cultural and religious practices. There are various methods used in the recycling of the autograft in the literature, but which method gives us the best incorporation of the graft with the host bone while retaining its biomechanical properties?

Currently, methods of sterilization described either in-vivo or in-vitro, include autoclaving [4,5], pasteurization [6-8], extracorporeal irradiation [9-12], microwave [13], and liquid nitrogen [14]. The main aims of sterilization of the recycled bone autograft are to eradicate the survival of tumour cells and yet maintain the important biological and structural properties of the bone graft.

The advantages of using recycled autograft are as follows (a) biological reconstruction with precise anatomical fit, (b) no donor site morbidity, (c) no risk of disease transmission such as HIV or other retroviral infections [15,16], (d) avoidance of immunological reaction, (e) avoidance of complications of prosthesis such as loosening, breakage, and wear [17,18], and (f) cheaper option compared to allografts as the latter requires a large scale bone bank system [19]. In addition, collagenous matrix and other intrinsic proteins seem to be preserved after irradiation, which may have contributed to the improved bony fusion and joint function of the osteoarticular grafts, compared with grafts processed by other methods [9-12]. An essential pre-requisite in recycling bone autograft is that the bone structure should be intact, as severely damaged bone would be too weak for skeletal reconstruction.

The disadvantages of recycled bone autografts are as follows (a) They can be mechanically weak and brittle depending on the sterilization process used, and this contributes to delayed union or non-union and fracture of the processed bone graft, (b) There is a potential risk of local recurrence due to tumour cell survival within the recycled bone autograft. The risk of local recurrence has been shown to be low as there are no viable tumour cells seen after different methods of sterilization [3].

At the moment, there is no study that looks at biological incorporation of the recycled bone to host bone. The present study focuses on three different methods of sterilization that are commonly used for treatment of bone autografts before reimplantation at our center. These are pasteurization, autoclaving and extracorporeal irradiation. This study is based on animal model (rabbit) and we wish to determine the most effective way to process the bone autografts without compromising the bone healing and structural properties. We will not be looking at tumour cells eradication as this is performed on a normal bone in an animal model. Tumour cell eradication has been proven by the author on resected tumour bones treated with different methods of sterilization (pasteurization, boiling, autoclaving and irradiation) [3].

## Methods

### Animals

Fully grown male New Zealand White rabbits (Oryctolagus cuniculus), weight 2-3 kg, were housed in standard cage at controlled temperature and humidity of 26°C and 60% respectively with free access to water and standard pellet food. Twenty-four animals were divided into 4 groups according to the methods of sterilization used to prepare the bone grafts including the control group. Two animals in each test group were sacrificed at 6^th^ weeks, 9^th^ weeks and 12^th^ weeks. The Animal Ethics Committee of this institution approved the animal study. All animals were maintained in accordance with a protocol approved by the Institutional Animal Care and Use Committee. All efforts were made to minimize animal suffering in this study.

### Surgery

The rabbits were anaesthetized with intramuscular injection of Ketamine (30 mg/kg IM) combined with Xylazine (3 mg/kg IM). General anesthesia was supplemented with 1% lignocaine injected subcutaneously at the operative site. The operation was carried out using aseptic technique at all times. A 6 cm longitudinal skin incision was made on the anteroproximal surface of the left lower hind leg of each rabbit. The periosteum and surrounding muscles were stripped off the diaphyseal cortex between the inferior edge of the tibial tuberosity and the tibiofibular synostosis. With an electric oscillating dental saw, a 5 cm long cylindrical segment of diaphysis measured from the inferior edge of the tibial tuberosity was removed. During the transverse open osteotomy, saline was poured onto the local bone to prevent thermal damage to the bone ends. After removal of the bone segment, the operation field was irrigated with saline and diluted povidine solutions. The rabbits were randomly assigned into one of the following four sterilization study groups [4-7,9,10]:

1. Group A – Control: Bone graft immersed in saline dish for 20 minutes (n = 6).
2. Group B – Pasteurization at 70°C for 20 minutes (n = 6).
3. Group C – Autoclaving at 135°C for 20 minutes (n = 6).
4. Group D – Irradiation with 50 Gy one fraction for 20 minutes (n = 6).

After sterilization, the treated tibial segment was reimplanted and fixed with an intramedullary 2.5 mm Kirschner wire. The surrounding soft tissues, the periosteum and the skin were carefully sutured. The Kirschner wire was cut short and embedded under the skin. The stability of the operated tibia was generally good and no external mobilization was applied. The rabbits were able to weight bear immediately on both hind legs. The surgical procedures were performed by a single surgeon to achieve a uniform technique. After surgery, all rabbits received antibiotic (Kombitrim 240 or Sulfamethoxazole 200 mg/Trimethoprim 40 mg, 1 ml/10 kg IM) and analgesia (Meloxicam 5 mg/ml, 0.3 ml/kg IM) for five days. No sign of infection or ambulation disturbance were observed during the experimental period.

Radiographs of the tibia were taken for all rabbits at 1^st^ week, 3^rd^ weeks, 6^th^ weeks, 9^th^ weeks and 12^th^ weeks after surgery. Two rabbits from each test group were euthanized by an overdose of pentobarbitone (90 mg/kg IV) at 6^th^ weeks, 9^th^ weeks and 12^th^ weeks. Both tibiae were harvested en bloc, cleaned of soft tissue, wrapped in a sterile drape, and kept frozen (-20°C) in airtight containers for subsequent histopathological analysis and biomechanical testing. We also recorded any gross union or non-union at the proximal and distal osteotomy sites before storing the bone specimens in the freezer.

### Tissue preparation

All the harvested bone specimens were stored at -20°C until testing. After thawing for 24 hours, 6 cm of tibial shaft was cut from the tibial tuberosity with an electric saw, then divided into three equal size specimens, each measuring 2 cm in length. The proximal segment was labeled as A, middle segment as B and distal segment as C. Segments A and C which contain the proximal and distal osteosynthesis sites were placed in labeled jar containing 10% neutral formalin solution for histopathological evaluation. Segment B was sent for biomechanical testing. All tissue specimens for histopathological evaluation were stained with haematoxylin and eosin (H&E).

### Methods of evaluation

#### Radiographic evaluation

Radiographs of the tibia (antero-posterior and lateral views) were taken at 1^st^, 3^rd^, 6^th^, 9^th^ and 12^th^ weeks after surgery. To semi-quantify the findings, the incorporation process as observed on the radiographs at each corner of the grafts was classified into five stages using the Takahashi score (1991) (Table 1) [7].

**Table 1:** **Takahashi score**

	Points
Non-union gap with no or only minimal callus formation	0
External or bridging callus formation	1
Visible resorption of the bone graft	2
The callus has remodelled into compact bone and more prominent than the bone graft	3
No visible bone graft	4

For each proximal and distal osteotomy sites, the two corners and mid-portions on each osteotomy site were scored on the antero-posterior and lateral views. Hence, a total of 12 portions were scored and summed for each graft with (maximum score of 48 points). This evaluation was performed by a radiologist who was blinded to eliminate bias.

#### Histopathology evaluation

Tissue morphology and cellular appearances of the healing process induced by implantation of autografts sterilized by different methods were analyzed. The histological features included in the evaluation are the restoration of continuity between the bone edges (union or non-union) and the type of callus formation at the bone edges. The presence of osteocytes within the bone graft and the percentage of lacunae occupied by osteocytes were taken as an indicator of the revitalization of the graft. The percentage of the area of bone marrow in the medullary cavity of the graft was calculated based on the presence of haemopoietic cells.

Even though histology clearly revealed differences in tissue reaction after different methods of sterilization, it was not possible to compare the status of specimens on the basis of descriptive histology. Thus, a semi-quantitative system was applied with numerical rates given for each morphological feature. Zoricic et al used this semi-quantitative system in his study on bone grafts in rabbits [7], and a blinded independent pathologist performed the microscopic histopathological evaluation (Table 2).

**Table 2:** **Shows the point system created to objectively evaluate the histological features of the specimens representing bone incorporation**

Points	0	1	2	3
Type of callus formation	no callus	fibrous	soft	hard
Area of lacunae occupied by osteocytes (%)	<25	25-50	50-75	>75
Area of bone marrow in the medullary cavity (%)	<25	25-50	50-75	>75

#### Biomechanical evaluation (compression test)

The 20-mm bone graft (segment B) was placed between two parallel stainless steel platens so that the long axis of the bone matched the compression axis using an Instron type 1026 mechanical testing machine (Instron Ltd, High Wycombe, UK). The compressive strength of the bone was measured with a speed of compression of 0.2 mm/s. The equipment was connected to a computer in order to determine the load deformation curve, and the maximum load was measured. For each sample, the test was interrupted once the first deflection of the stress/strain curve was obtained. Deformation at the time of failure was measured, and elasticity modulus was calculated in the first, straight section of the strain deformation curve. The same strength test was performed for all groups, and the data were analyzed using one-way ANOVA, and post-hoc comparisons of means.

All histomorphometric data are given as mean values ± standard error of the mean. Statistical analysis was performed using the Student’s *t*-test and P values of < 0.05 were considered statistically significant. SPSS version 14 was used to analyze the data.

## Results

### Radiographic findings

**Figure 1:**
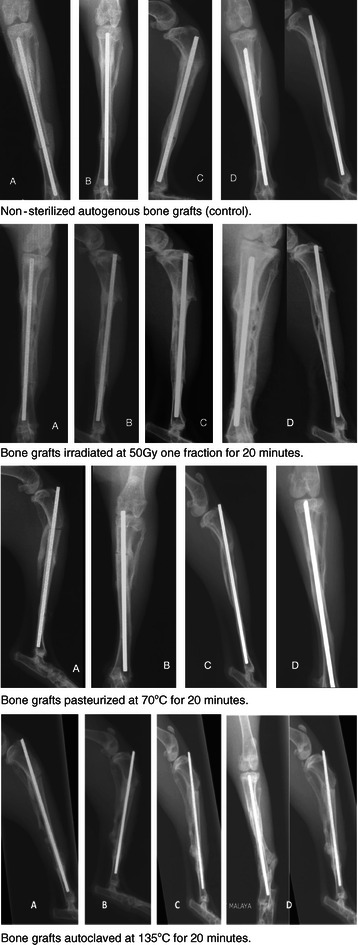
Post-operative radiographs of various form of sterilization for the different groups showing different stages of bone union seen at weeks 3 till weeks 12. At: 3 weeks **(A)** – bridging callus formation at the osteotomy sites. 6 weeks **(B)** – more callus formation seen 9 weeks **(C)** – hardening of callus and disappearance of osteotomy lines 12 weeks **(D)** – solid union of the bone grafts.

Radiographs of the 4 different methods of sterilization are shown in Figure 1. In the control group, at 3 weeks after reimplantation, the grafted segment had already been firmly fixed with external callus bridging over the osteotomy gaps. The progress of callus formation and bone resorption can be seen at 6 and 9 weeks. At 12 weeks, graft resorption was observed in more extensive areas, while the callus remodeled to form new compact bone. Although grafts had been resorbed to various degrees, most of the proximal and distal ends of the grafts were still distinguishable on the anterior-posterior and lateral views.

**Figure 2:**
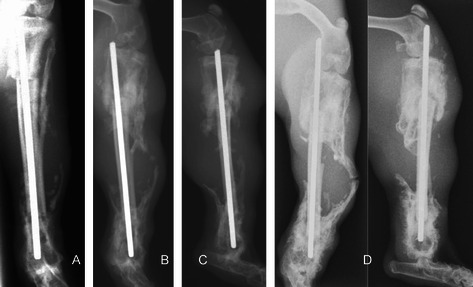
This rabbit has unusual reaction to the autoclaved bone graft. Radiographs at 3 weeks **(A)**, 6 weeks **(B)** and 9 weeks **(C)** showed abundant external callus formation and bone resorption at proximal and distal osteotomy sites. Radiographs at 12 weeks **(D)** showed extensive bone graft resorption and this caused shortening of the tibia, proximal migration of the K-wire and bending of the fibula.

Grafts treated with irradiation at 50 Gy generally showed a similar pattern on incorporation. Similar findings were also noted in the pasteurization group. Both irradiation and pasteurization groups have a good callus formation and bony union at the osteotomy sites. The autoclave group had poorer radiological results. One of the rabbits in this group had unusual reaction. There was considerable bone resorption at the osteotomy sites mainly involving the normal host bone rather than the graft itself (Figure 2). There was also abundant callus formation seen at the proximal and distal osteotomy sites. In spite of the deformed and shortened left leg, the rabbit was well till the end of the study period with no signs of infection at the operated leg.

**Figure 3:**
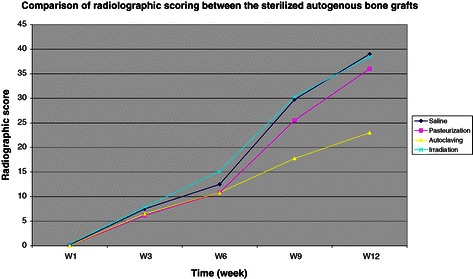
Chart showing the radiographic scoring between the sterilized bone grafts. All groups show increasing score pattern which is in keeping with bone healing or incorporation process over the period of 12 weeks until solid bone union. This chart showed that irradiation and pasteurization techniques are significantly better than autoclaving technique in preserving the bone healing of the recycled bone grafts.

Table 3 and Figure 3 show the results of semi-quantitative evaluation of the radiographs using the Takahashi scoring system. Increase in score numbers over time was in keeping with the incorporation process as observed qualitatively on radiographs. At 9 and 12 weeks, the autoclaving group scored a slightly lower average of points than the control group and the other two sterilized groups. The irradiation group has a better radiographic score than the pasteurization and autoclaving groups, and it is also closely related to the control group.

**Table 3:** **Semi-quantitative radiographic evaluation: descriptive data**

Weeks post-op	Saline	Pasteurization	Autoclaving	Irradiation
1 (n = 6)	0.2 ± 0.4	0	0	0.3 ± 0.5
3 (n = 6)	7.5 ± 2.3	6.2 ± 1.5	6.5 ± 1.6	7.8 ± 0.8
6 (n = 6)	12.5 ± 2.7	10.8 ± 1.9	10.8 ± 1.3	15.2 ± 3.7
9 (n = 4)	29.8 ± 2.1	25.5 ± 2.5	17.8 ± 2.2	30.3 ± 3.9
12 (n = 2)	39 ± 2.8	36 ± 8.5	23 ± 2.8	38.5 ± 2.1

Note: Data are means ± standard deviation. () = Number of samples in each group.

As the data is normally distributed, one-way ANOVA test was used. There are significant differences between the treatment groups at week 6 (p = 0.028) and week 9 (p = 0.000) post-operatively.

Further statistical analysis shows that there are no significant differences seen between all the groups at the initial stage of 1 week (p = 0.817) and 3 weeks (0.984) postoperatively. At 6 weeks, the irradiation group has significantly (p = 0.041) higher score than pasteurization and autoclaving groups. At 9 weeks, the autoclaving group has significantly (p = 0.000) lower score than the other groups and this is illustrated clearly in Figure 3. At 12 weeks, the irradiation group has higher score than pasteurization and autoclaving groups but this was not statistically significant (p = 1.000).

### Biomechanical test results

**Figure 4:**
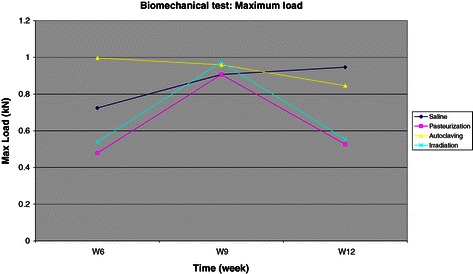
Chart for maximum load under compression test.

**Figure 5:**
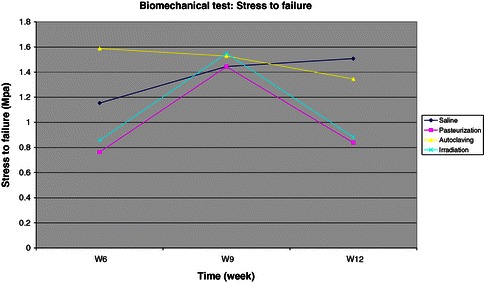
Chart for stress to failure under compression test. Shows similar pattern seen in the maximum load under compression test (see Figure 4). The control group is increasingly getting stronger towards the end of the study at week 12 as the grafts become incorporated and united. All sterilized bone grafts become weaker towards the end of the study at 12 weeks compared to the control group.

Biomechanical test was performed on the bone grafts as compression test to simulate the axial loading on the tibia on weight bearing based on the maximum load, stress to failure and strain to failure under compression test. The results showed that autoclaved bone graft generally has a higher maximum load (kN) (Figure 4), stress to failure (Mpa) (Figure 5) and strain to failure (%) compared to the other two groups. But this was not statistically significant at 6 (p = 0.389), 9 (p = 0.999) and 12 weeks (p = 0.259). In all sterilized bone graft groups, the mechanical strength drop significantly compared to the control group at 12 weeks, which shows that the graft is weakened over time. Pasteurized autogenous bone graft has the lowest mechanical strength throughout the compression test.

In summary, all sterilized bone graft mechanical strength drop from the three different biomechanical tests. We found that the autoclaved autogenous bone graft has the highest mechanical strength compared to the control group, followed by the irradiation group and pasteurization group. The autoclaved bone graft was weakened by 10-16% whilst the irradiated and pasteurized bone grafts were weakened by 40-67% compared to the control group. This finding is unexpected as we expected the autoclaved bone graft would perform the poorest in all biomechanical tests. Heat treatment higher than 100°C on the bone causes degeneration of the bone collagen, thus causing decrease in the bone mechanical strength. This variation in results may be due to the limitation of bone graft length taken from the diaphysis and not from the whole tibia. The full potential of bone graft strength was not tested in this study.

### Histological analysis

#### Macroscopic evaluation

Union at the osteotomy sites was assessed clinically after we harvested the tibia at the end of 6^th^, 9^th^ and 12^th^ weeks postoperatively. Percentage of bony union was calculated by number of union at proximal and distal osteotomy sites divided over the total number of union in each group. The total number of union in each group will be 12, as each group has 6 rabbits and each rabbit has 2 osteotomy sites. The results are shown in Table 4.

**Table 4:** **Shows union at the various sites in the various groups**

Union at osteotomy sites in control group (n = 6)			
Site of union	W6	W9	W12
Proximal & Distal	1	2	2
Proximal	1	0	0
Distal	0	0	0
None	0	0	0
Union at osteotomy sites in pasteurization group (n = 6)			
Site of union	W6	W9	W12
Proximal & Distal	1	0	1
Proximal	1	2	1
Distal	0	0	0
None	0	0	0
Union at osteotomy sites in autoclaving group (n = 6)			
Site of union	W6	W9	W12
Proximal & Distal	2	2	1
Proximal	0	0	0
Distal	0	0	0
None	0	0	1
Union at osteotomy sites in irradiation group (n = 6)			
Site of union	W6	W9	W12
Proximal & Distal	1	2	2
Proximal	1	0	0
Distal	0	0	0
None	0	0	0

In the control group, we had one rabbit with non-union at the distal osteotomy site, and the rest has a complete union. The percentage of bony union was 91.7%. This is unexpected as the graft was not treated with any sterilization technique and there were no signs of infection.

Pasteurization has the highest rate of non-union, as only 2 rabbits have a complete bony union, whereas the other 4 rabbits have a non-union at the distal osteotomy site. The percentage of bony union was 66.7%. Autoclaving group has one complete non-union and the others have a complete union at the proximal and distal osteotomy sites. The percentage of bony union was 83.3%. Irradiation group has a similar rate of union and non-union as the control group with only one non-union at the distal osteotomy site. The percentage of bony union was 91.7%. There is higher distal osteotomy non-union rate, which is non uncommon in distal third tibial fracture healing due to poor vascularity. Non-union in a recycled bone graft is a known complication due to destruction of collagenous matrix and intrinsic proteins during sterilization process [5,6,9,10,15]. From the macroscopic evaluation, the irradiation group appears to have a better overall union rate compared to the pasteurization and autoclaving groups.

### Microscopic evaluation

A semi-quantitative system was created with numerical rates for each morphological structure to give an objective assessment of microscopic union (Table 5).

**Table 5:** **Summary of semi-quantitative analysis of the histopathological features in the bone grafts after sterilization at 6** ^**th**^ **, 9** ^**th**^ **and 12** ^**th**^ **weeks of study**

Group	Type of callus		
Weeks	6th	9^th^	12th
Saline (n = 6)	12	15	18
Pasteurization (n = 6)	12	9	14
Irradiation (n = 6)	11	14	11
Autoclaving (n = 6)	14	15	6
**Group**	**Osteocytes (%)**		
**Weeks**	**6th**	**9th**	**12th**
Saline (n = 6)	14	15	16
Pasteurization (n = 6)	16	14	15
Irradiation (n = 6)	12	13	10
Autoclaving (n = 6)	14	15	5
**Group**	**Bone marrow (%)**		
**Weeks**	**6th**	**9th**	**12th**
Saline (n = 6)	4	6	2
Pasteurization (n = 6)	6	5	9
Irradiation (n = 6)	13	12	8
Autoclaving (n = 6)	12	11	1

At the end of 12^th^ weeks, pasteurization group scored the highest points in callus formation, area occupied by osteocytes and area of bone marrow in the medullary cavity as compared to the other two sterilization groups. This was only statistically significant at the 12^th^ weeks (p = 0.041), but not at the 6^th^ (p = 0.685) and 9^th^ weeks (p = 0.310) of study. The autoclaved group had the lowest points in all categories especially at the end of 12^th^ weeks, which was statistically significant (p = 0.009).

## Discussion

As we know the gold standard for limb salvage surgery is endoprosthesis replacement or the use of allograft. But sometimes due to limited resources, these things are not at the surgeon’s disposal and the surgeon has to improvise with whatever method is cost effective in order to avoid an amputation. Recycling of the a strelize tumour bone as a bone graft, is a feasible method of reconstruction in centers with limited resources as its an inexpensive method of limb salvage. This study is conducted to determine the best method of sterilization that gives 100 percent tumour kill with maximum incorporation into host bone.

The present study focuses on three different methods of sterilization that are available at our centre and commonly used for treatment of autogenous bone grafts before reimplantation. These are pasteurization, autoclaving and irradiation (extracorporeal radiotherapy). Liquid nitrogen was not tested in this study because we do not have the necessary facilities to handle the chemical. This experimental study shown that both irradiation and pasteurization techniques have more favorable outcome in terms of bony union based on radiographic and macroscopic histopathological evaluations. Pasteurization has the highest score in microscopic histopathological analysis, which make it superior than irradiation. Autoclaving did not perform well in both radiographic and histological evaluations. Biomechanically, all grafts will be dramatically weakened over time after sterilization and this is shown from our study.

Among various kinds of bone grafts, fresh autogenous bone grafts incorporate the fastest. The normal incorporation process of autogenous bone grafts is supported by the contribution of graft derived cells, local growth factors stored in the matrices including bone morphogenic protein and transforming growth factors, and also structural properties of matrices such as osteoconductive and piezoelectric properties [20,21].

The incorporation process of normal cortical bone graft has been documented by a number of authors [8,20,22-24]. The graft incorporation of cancellous bone begins immediately with apposition of new bone to dead trabeculae but for the cortical bone, it is initiated by resorption, followed by delayed formation of new bone. In cortical bone grafts in dogs, initial resorption causes porosity, which begins as early as 2 weeks after implantation and peaks around 6 months [22]. Although new bone formation follows, the graft remains a mixture of dead and viable bone, and remodeling continues for years.

Our observation of bone healing over 12-week period in rabbits followed a different order of events. The initial reaction of local tissues to operation was predominantly new bone formation at the osteotomy sites, followed by coupling of bone resorption and formation. These events were seen in all groups. This discrepancy is due to several factors; first, the rabbit is an animal of higher metabolic activity that the larger species such as dog; second, a large part of periosteum was conserved during the operation; third, fixation of segmental osteotomy with an intramedullary wire is not of rigid fixation, thus allows some degree of movement at the junction, facilitating formation of external callus; fourth, the volume of the graft was relatively small, so that the early repair reactions that are normally confined to marginal portions could cover the entire graft much faster; fifth, the rabbits in our experiment were bearing weight on the operated tibia, so that bone formation was augmented on the basis of Wolff’s law; and lastly, the part of tibial diaphysis resected in the present experiment is composed solely of cortical bone and fatty bone marrow, and is completely devoid of trabecular bone.

As seen in our results, all the grafts were considered united at 12^th^ weeks based on the radiographic analysis and this was seen by the increasing trend in the scoring given. The incorporation process in irradiated bone autograft is as good as in the control group and statistically significant compared to the other techniques. Autoclaving has a slowest incorporation process, likely due to denaturation of important local growth factors including bone morphogenic proteins in the matrices.

During the macroscopic evaluation, there were actually more non-union than expected from the radiographic analysis. The non-union predominantly occurred at the distal osteotomy site, 6 out of 24 rabbits (25%), and four rabbits are from the pasteurization group. We only had one complete non-union from the autoclaving group and the same rabbit has abundant external callus formation and severe bone resorption seen on the radiographs. Irradiation group has a similar rate of union and non-union as the control group with only one non-union at the distal osteotomy site. The percentage of bony union was 91.7%. From the macroscopic evaluation, the irradiation group appears to have a better union rate compared to the pasteurization and autoclaving groups. However, in microscopic evaluation at the end of 12^th^ weeks, pasteurization group scored the highest points in callus formation, area occupied by osteocytes and area of bone marrow in the medullary cavity as compared to the other two sterilization groups. Autoclaving group has the lowest points in all categories. Microscopic evaluation is a better determinant in defining true union and bone grafts viability compared to bone incorporation based on radiographic appearance. Microscopic analysis looks at osteoid formation, area occupied by osteocytes and bone marrow cellularity, which are important for long-term bone graft union at graft-host junction. Thus, pasteurized bone graft performed well in all three categories in microscopic analysis showing a good potential for further bone union and remodeling.

The sterilization process in this study used two levels of temperature at 70°C in pasteurization and 135°C in autoclaving. It is known that the inductive capacity of bone is reduced with increasing temperature and increasing duration. An increase of temperature between 80°C to 134°C is reported to reduce healing [25]. Thus irradiation and pasteurization groups appear to have more favorable outcomes than autoclaving group as shown in the radiographic analysis as well as in the histopathological analysis. The osteoinductive and osteogenic capacity of the bone is largely destroyed in autoclaving due to very high temperature [26-30]. Irradiated bone autografts seems to be better in the union rate than the pasteurized bone autograft based on radiological and biomechanical tests. However, microscopic histological analysis showed that pasteurized bone grafts preserved the osteocytes and bone marrow better. This microscopic finding is important to determine the true union at the osteotomy sites and potential for further bone healing. Uyttendaele suggested that irradiation seems to preserve important collagenous matrix and other intrinsic proteins, which may have contributed to the improved bony fusion and joint function of the osteoarticular grafts, compared with grafts processed by other methods [31]. Nobuhito Araki et al from Osaka Medical Center, Osaka, Japan have used irradiation for the reconstruction of bony defects in bone and soft tissue tumour surgery since 1989 and reported the clinical results of 20 patients, including their radiologic findings, functional analyses, and complications [9]. He found that radiologically, bony union occurred in 23 out of 29 (79%) osteotomy sites. The overall radiographic evaluation rating was 74%. Nonunion (20%) and infection (15%) were the two major complications. Similarly, few other authors [11,12,32-34] have shown successful union of pasteurized and irradiated bone autografts in their series of skeletal reconstruction after tumour resection. These results indicate that both, extracorporeal irradiation or pasteurization can be applied for reconstruction surgery after tumour resection.

There are several papers reporting the effect of heat treatment itself on the bone in regard to mechanical strength changes [25,29,34]. Köhler et al reported in a study using the diaphyseal bone of rabbits that the strength decreased to 77% in a torsional test after being autoclaved at 121°C for 20 min [29]. Knaepler et al. reported in his study using pig cancellous bone that the compressive strength decreased to approximately 60% after 100°C treatment, but the mechanical strength was not influenced at 60°C of heat treatment [25]. In our study, the results showed that in all sterilized bone graft groups, the mechanical strength drop significantly up to 67% compared to the control group at 12^th^ weeks, which shows that the graft is weakened over time. The biomechanical performance of all grafts decreased steadily after 9^th^ weeks. Pasteurized bone autografts has the lowest mechanical strength throughout the compression test. The cause of this biomechanical degradation in all groups is likely due to damage of the bone microstructure due to heating. It is known that bone collagen attributes to the bone strength, and that its properties are changed by heating. Vangsness et al. reported that collagen structure changes at temperatures higher than 80°C [35]. While Urist et al. reported that bone collagen did not shrink with temperatures below 60°C [36]. These reports suggest that bone collagen degenerated at 100°C heat treatment, causing a decrease in the mechanical strength, while heat-treated bone below 60°C was not affected. This study showed that extreme heat treatments should be avoided, especially if it is more than 100°C. Although the bone autografts strength gradually decreases with time, it acts as structural framework for bone growth in achieving complete union and remodeling. It is expected that new bone will continue to form within the autografts beyond three months, which is not observed in this study.

## Conclusion

This experimental study shown that both irradiation and pasteurization techniques have more favorable outcome in terms of bony union based on radiographic and macroscopic histopathological evaluations. Pasteurization has the highest score in microscopic histopathological analysis, which make it superior than irradiation. Autoclaving did not perform well in both radiographic and histological evaluations. Biomechanically, all grafts will be dramatically weakened over time after sterilization and this is shown from our study. Although both irradiation and pasteurization methods offer a simple way to sterilize bone autografts, we feel pasteurization offers the best overall outcome and can be a useful option to reconstruct a large bone defect after tumour resection.

### Limitations of the study

The small size of bone specimen resected in the middle part of the tibial diaphysis for biomechanical analysis may not be representative of the whole tibia and these may affect the result of the mechanical test. The small number of animals per group and a short duration of study have an effect on the power of analysis in this study. As there are two animals sacrificed at the successive time points, there is low number of animals at the end of the study. Thus we are not able to follow the full potential of union for each animal as each individual response might vary to each sterilization techniques. We were only able to study small number of animals and conducted it for 3 months due to limited funding and resources. A larger sample size and longer duration of study would give a more reliable outcome of the graft after each sterilization process.
